# Disulfide-mediated conversion of 8-mer bowl-like protein architecture into three different nanocages

**DOI:** 10.1038/s41467-019-08788-9

**Published:** 2019-02-15

**Authors:** Jiachen Zang, Hai Chen, Xiaorong Zhang, Chenxi Zhang, Jing Guo, Ming Du, Guanghua Zhao

**Affiliations:** 1 Beijing Advanced Innovation Center for Food Nutrition and Human Health, College of Food Science & Nutritional Engineering, China Agricultural University, Key Laboratory of Functional Dairy, Ministry of Education, 100083 Beijing, China; 2 Center of Biomedical Analysis, Tsinghua University, 100084 Beijing, China; 3 School of Food Science and Technology, National Engineering Research Center of Seafood, Dalian Polytechnic University, 116034 Dalian, China

## Abstract

Constructing different protein nanostructures with high-order discrete architectures by using one single building block remains a challenge. Here, we present a simple, effective disulfide-mediated approach to prepare a set of protein nanocages with different geometries from single building block. By genetically deleting an inherent intra-subunit disulfide bond, we can render the conversion of an 8-mer bowl-like protein architecture (NF-8) into a 24-mer ferritin-like nanocage in solution, while selective insertion of an inter-subunit disulfide bond into NF-8 triggers its conversion into a 16-mer lenticular nanocage. Deletion of the same intra-subunit disulfide bond and insertion of the inter-subunit disulfide bond results in the conversion of NF-8 into a 48-mer protein nanocage in solution. Thus, in the laboratory, simple mutation of one protein building block can generate three different protein nanocages in a manner that is highly reminiscent of natural pentamer building block originating from viral capsids that self-assemble into protein assemblies with different symmetries.

## Abstract (web summary)

Shape transformation of proteins created by design in the laboratory is challenging. Here, the authors present a disulfide-mediated approach for the preparation of 16-mer, 24-mer, and 48-mer nanocages from an 8-mer bowl-like protein building block.

## Introduction

Shape transformation is a popular phenomenon in nature, by which living organisms perform shape-to-function activities in response to the external environment^1–5^. Proteins are nature’s most versatile building blocks, programmed at the genetic level to perform myriad functions and are largely responsible for the complexity of an organism^6^. In viral capsids, a single protein fold can be evolved to form multiple oligomeric states with different symmetries^7^, but the shape transformation of proteins created by design in the laboratory has largely been inaccessible. Generally, noncovalent interactions are mainly involved in the formation of the protein quaternary structures where subunit–subunit interactions (SSIs) are involved^8–10^. Such noncovalent interactions at subunit–subunit interfaces are exquisitely controlled, which define the geometry of protein architectures. Although the protein architectures are usually governed by noncovalent interactions at the subunit−subunit interfaces, the energetic contributions of individual residues to the stability of subunit−subunit interfaces are often unevenly distributed^11,12^. To find the key individual residues responsible for SSIs could provide a solution to control the conversion of one quaternary structure into another^13^.

Similarly, disulfide bonds likewise play an important role in the formation and stability of proteins^14^. Disulfide bonds existing in proteins are relatively oxidative in the extracellular space^15^. Recently, disulfide-functionalized nanoparticles and organic polymer hydrogels have been rapidly developed as delivery carriers;^16^ moreover, disulfide bonds have been exploited as bridges to construct 2D and 3D protein nanomaterials^17,18^. However, the function of disulfide bonds in the conversion between different protein architectures and in the fabrication of protein nanocages has yet to be explored.

Protein cages are widely distributed in nature to fulfill a variety of functions^19^, which usually have highly symmetrical structures constructed from versatile building blocks. Self-assembled protein nanocages represent a class of nanoscale scaffolds that holds much promise for various applications^19–25^. However, the number and structure of naturally occurring proteins are limited, thereby impeding their further applications as biotemplates or vehicles in the field of nanoscience and nanotechnology. To overcome this limitation, different methods, including the matching rotational symmetry approach^26,27^, computational interface design^28,29^, and directed evolution have been explored to create different protein cages^20^, but these approaches are usually engineering-intensive for protein surface and highly dependent on the accuracy of the design, thereby negatively impacting the biological activity of the designed protein.

To address these issues, we try to use a simple chemical-bonding approach to control SSIs, thereby constructing protein architectures with minimal design. We believe that cysteine (Cys)-mediated disulfide bonds fit this approach well because (1) they are strong and reversible, and such properties can minimize the surface area to be designed, while keeping them chemically tunable; and (2) they are easily designed and engineered by well-established chemical and genetic techniques.

**Fig. 1:**
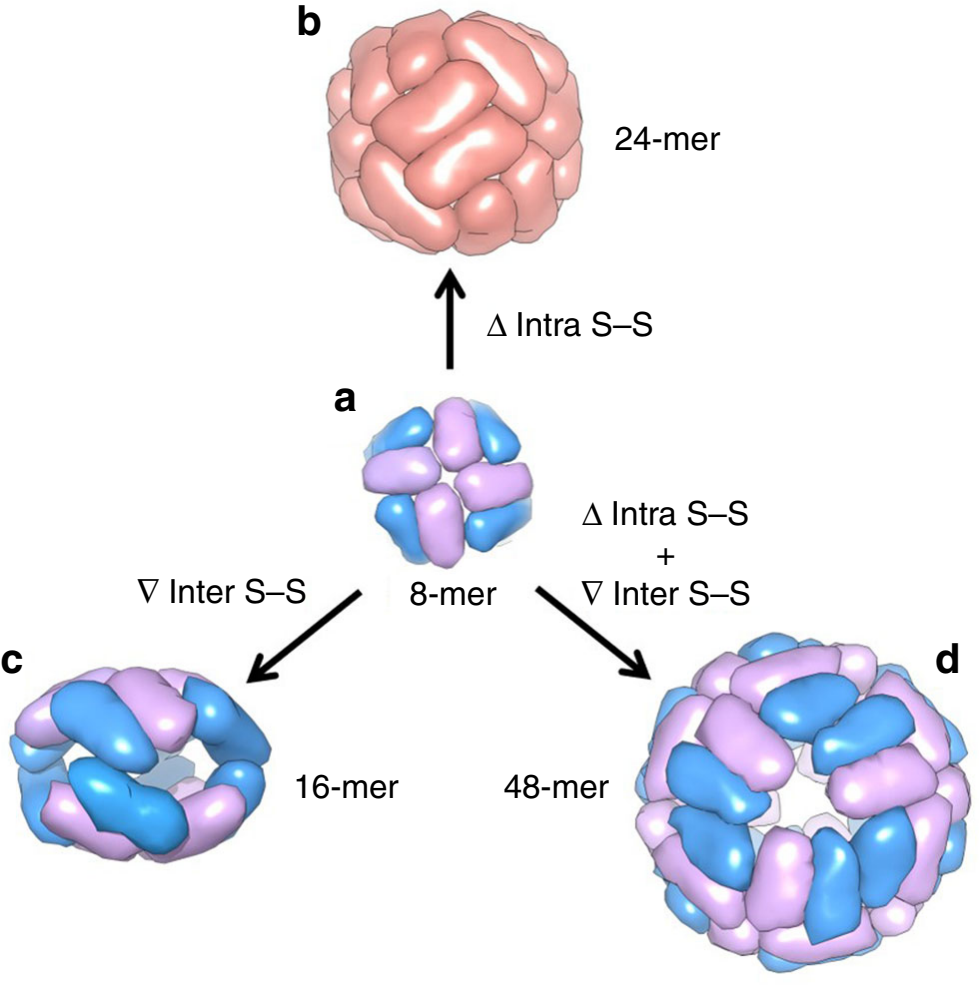
Schematic representation of the conversions from 8-mer bowl-like proteins. **a** NF-8 as a building block is an 8-meric heteropolymer composed of H_α_ (blue) and H_β_ (purple) subunits at a ratio of 1:1. **b** Deletion of intra S–S bond resulted in the conversion of NF-8 into a 24-mer ferritin-like protein nanocage that is composed of the H_γ_ subunit (red). **c** Insertion of inter S–S bonds led to the conversion of NF-8 into a 16-mer protein nanocage containing H_α_ and H_β_ subunits. **d** Deletion of the same intra S–S and insertion of the inter S–S bonds caused the conversion of NF-8 into a 48-mer protein nanocage also consisting of H_α_ and H_β_ subunits

Herein, we report a set of discrete protein nanocages with different sizes and geometries (24-mer, 16-mer, and 48-mer), which are constructed by using one single 8-mer bowl-like protein building block through deletion of one inherent intra-subunit S–S bond formed within one subunit, insertion of inter-subunit S–S bonds at the protein interface, and deletion of the intra-subunit S–S bonds while insertion of the inter-subunit S–S bonds, respectively (Fig. 1). This disulfide-mediated approach to the conversion between different protein assemblies opens up an avenue for protein assemblies with unexplored properties.

## Results

### Conversion of 8-mer architecture into 24-mer nanocage

As a standard structural component among protein nanocages, ferritin exists ubiquitously in both prokaryotes and eukaryotes. It is a nearly spherical 24-subunits protein with an exterior diameter of about 12 nm and a hollow cavity of 8 nm^30,31^. Owing to its cage-like morphology and highly symmetrical structure, ferritin has been explored as a nanocarrier for the preparation of different nanomaterials^19,22–24,32^. Nevertheless, so far, the ferritin assembly has been limited in scope to a single size and shape.

**Fig. 2:**
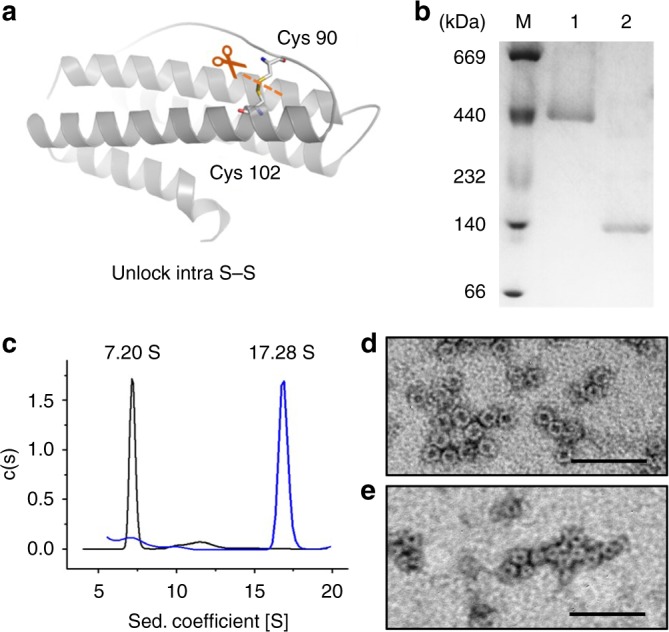
Deletion of intra S–S converts NF-8 into two protein assemblies. **a** Structural design of the H_α_ subunit of NF-8 that contains an intra S–S bond. **b** Native PAGE of two overexpressed products related to mutant Δ3C. Lane 1 corresponds to the protein species with the larger MW, while lane 2 represents the protein species with the smaller MW. Source data are provided as a Source Data file. **c** Sedimentation coefficient distribution of the two products. **d** TEM image of the protein product with the larger MW. **e** TEM image of the protein product with the smaller MW. Scale bars represent 50 nm

Recently, by introduction of small (hexapeptide) deletion into helix D of each subunit^13^, we carried out the complete conversion of native 24-mer ferritin nanocage into a 8-mer bowl-like non-native protein architecture in solution (Supplementary Figure 1), and this protein was referred to as NF-8. This fabricated protein assembly is stable in different buffer solutions over the pH range of 6.0–9.0, and thus it has great potential as a building block to construct protein architectures^13^. Structurally, the 8-mer protein is a heteropolymer that is composed of two different subunits (H_α_ and H_β_) that originate from the same polypeptide. During its self-assembly process, these two subunits form a dimer with a ratio of 1:1, and then four of them assemble into an 8-mer protein architecture with *C*_*4*_ symmetry^13^. Notably, there is an intra-subunit disulfide bond (Cys90–Cys102) formed within each H_α_ subunit of NF-8, while the H_β_ subunit is devoid of such disulfide bond. Structural analyses reveal that Cys90 in the BC loop is far away from Cys102 located at the C-helix in the H_β_ subunit or native HuHF subunit, but these two cysteines are in close proximity in the H_α_ subunit and thus form an intra-subunit S–S bond that causes an obvious shift of the C-helix to the direction of the B-helix, while D-helix is moving to the opposite side (Supplementary Figure 2). We envisioned that this intra-subunit disulfide bond could play an important role in maintaining the tertiary structure of the H_α_ subunit, thereby stabilizing the quaternary structure of NF-8. To test this idea, we planned to delete this intra disulfide bond by genetic modification (Fig. 2a), and then observed the possible structural changes due to such deletion. To this end, we made a mutant named Δ3C, where two cysteine residues (Cys90 and Cys102) related to the formation of the intra-subunit S–S and another free cysteine (Cys130) were replaced by alanine (Ala), respectively (Supplementary Figure 3). Cys130 was removed to just prevent the production of any possible inclusion body through incorrect disulfide bond linkage. After *E. coli* cells expressing the proteins grew at 20 °C for 8 h, the resulting proteins were analyzed by native PAGE. The yield of this mutant is about 40 mg per 1 L of culture medium under the present experimental conditions, which is similar to that of recombinant human H chain ferritin (HuHF). The results showed that there are two overexpressed species, namely one major species with a larger molecular weight (MW) and another minor species having a smaller MW. In contrast, when the expression of mutant Δ3C in *E. coli* was carried out at 37 °C, the yield of these two protein species is reversed as shown in Supplementary Figure 4.

To gain insight into the nature of these two protein species, both of them were purified by a combination of gel and ion-exchange chromatography, followed by characterization. Native PAGE of these two proteins showed a single band (Fig. 2b), indicating that they were purified to homogeneity. Analytical ultracentrifugation showed that the larger protein assembly in solution sedimented as a single discrete species with s_20,w_ = 17.28 S (Fig. 2c), which is very similar to that of wild-type (wt) HuHF (s_20,w_ = 18.8 S)^13^, suggesting that it is also a 24-mer protein assembly, so it is referred to as 24-mer_Δ3C_. In contrast, the smaller species was sedimented to obtain as s_20,w_ = 7.20 S, which is nearly the same as that of NF-8 (s_20,w_ = 7.4 S)^13^, suggesting that it is also a 8-mer protein assembly, and is termed as 8-mer_Δ3C_. Consistent with the above conclusion, transmission electron microscopy (TEM) analyses revealed that the exterior diameter of 24-mer_Δ3C_ is about 12 nm, which is almost identical to that of native ferritin, while 8-mer_Δ3C_ has an exterior diameter of ~9 nm, a value being the same as the size of NF-8^13^.

**Fig. 3:**
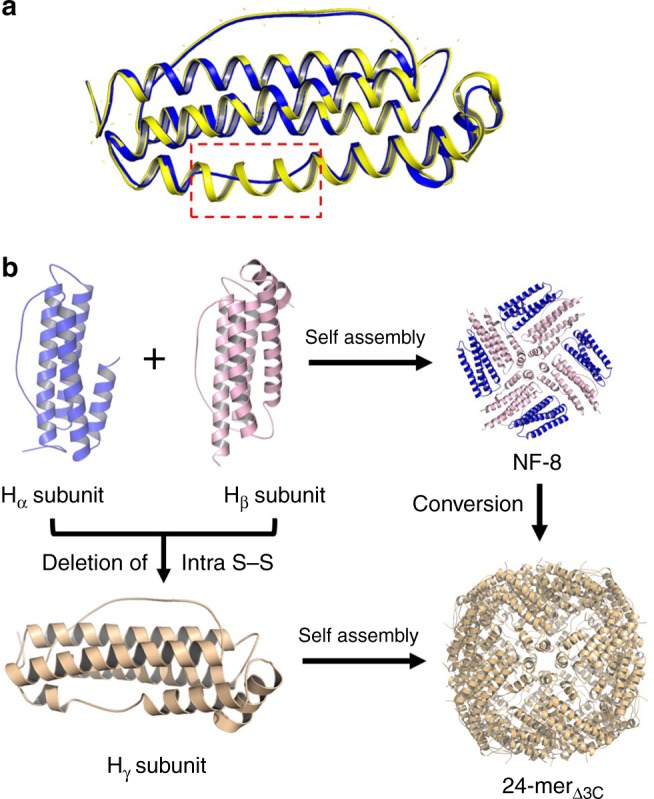
The crystal structure of 24-mer_Δ3C_. **a** Superposition of 24-mer_Δ3C_ H_γ_ subunit (blue) and native HuHF subunit (yellow) revealed that a shortage of α-helix in the middle of the D-helix of native HuHF subunit is replaced by a loop in the H_γ_ subunit. **b** Deletion of the intra S–S bond of NF-8 resulted in the conversion of H_α_ and H_β_ subunits into the H_γ_ subunit, 24 of which assemble into a 24-mer protein nanocage, 24-mer_Δ3C_. This corresponds to the conversion mechanism of NF-8 to 24-mer_Δ3C_

To obtain detailed structural information on 24-mer_Δ3C_, we tried to crystallize this protein and eventually obtained qualified crystals suitable for X-ray diffraction. We solved the crystal structure at resolution of 3.104 Å (Supplementary Tables 1 and 2). We found that 24-mer_Δ3C_ is composed of 24 subunits assembling into a ferritin-like nanocage (Supplementary Figure 4), approving the above hypothesis that the deletion of the intra-subunit S–S bond of NF-8 leads to such conversion. The structural analyses revealed the large difference in structure between NF-8 and 24-mer_Δ3C_. NF-8 has the *C*_*4*_ symmetry, while 24-mer_Δ3C_ has an octahedral symmetry, so this fabricated 24-mer protein has the three *C*_*4*_, four *C*_*3*_, and six *C*_*2*_ rotation axes (Supplementary Figure 5). This represents the first structural difference for these two proteins. It was also observed that the orientation of the side chain of Cys130 in NF-8 is nearly the same as that of Ala130 in 24-mer_Δ3C_ (Supplementary Figure 6), indicating that the above mutation of Cys130 into Ala almost has no effect on the conversion of NF-8 into 24-mer_Δ3C_. The second difference in structure between NF-8 and 24-mer_Δ3C_ is that NF-8 is a heteropolymeric protein containing equal numbers of H_α_ and H_β_ subunits, while 24-mer_Δ3C_ is a homopolymer which consists of 24 identical subunits. Notably, the structure of 24-mer_Δ3C_ subunit differs strikingly from that of H_α_ and H_β_ subunits, and thus it is named as H_γ_ which also forms a four-α-helix bundle just like the subunit of native HuHF as shown in Fig. 3a. This might be an important reason why 24 H_γ_ subunits in 24-mer_Δ3C_ are able to assemble into a ferritin-like hollow structure. However, the superposition of H_γ_ and native HuHF subunits revealed that a shortage of an inherent α-helix in the middle of the D-helix is lost in the H_γ_ subunit (Fig. 3a). Consistent with this structural difference, we found that the stability of 24-mer_Δ3C_ is lower than that of wt ferritin, namely, 24-mer_Δ3C_ can dissociate into subunits at pH 3.0 (Supplementary Figure 7), while wt HuHF disassembly requires at least pH 2.0^25^. Thus, the deletion of a single inherent intra-subunit S–S triggers the conversion of H_α_ and H_β_ subunits of the NF-8 protein architecture into their H_γ_ analog, 24 of which, therefore, assemble into a 24-mer protein nanocage (Fig. 3c), this corresponding to the possible conversion mechanism of NF-8 into 24-mer_Δ3C_.

**Fig. 4:**
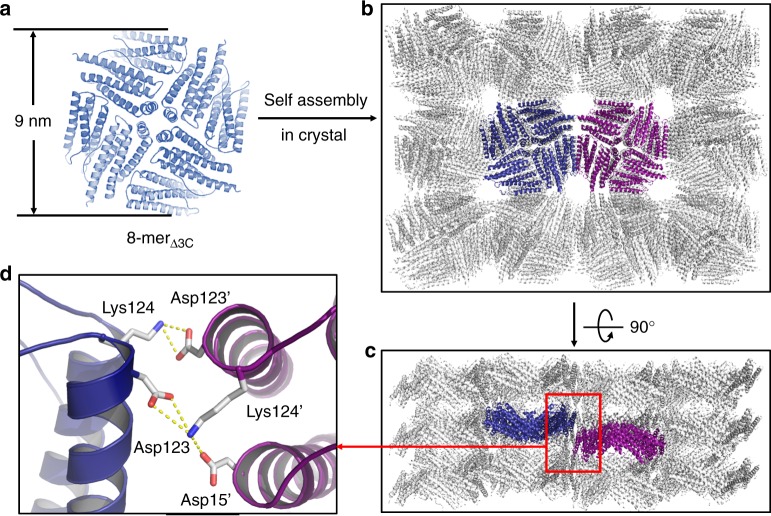
The crystal structure of 8-mer_Δ3C_. **a** The crystal structure of 8-mer_Δ3C_ is consistent with that in solution. This protein has a bowl-like structure with an outer diameter of 9 nm. **b** From the top view, 8-mer_Δ3C_ molecules assemble into 3D porous protein assemblies in the crystal. **c** From the side view, bowl-like 8-mer_Δ3C_ molecules array to form 2D protein layers. **d** Two salt bridges are formed between two adjacent 8-mer_Δ3C_ molecules having opposite orientations

**Fig. 5:**
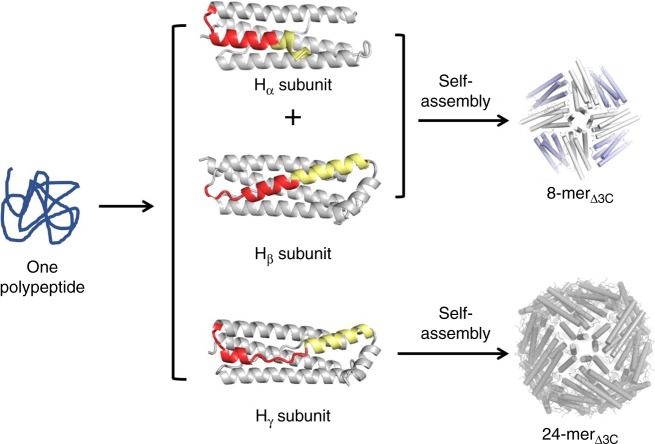
The construction of two proteins through different mechanisms. One polypeptide of mutant Δ3C folds into three kinds of subunits H_α_, H_β_, and H_γ_, and their difference in structure is highlighted in red and yellow; subsequently, the first two types of subunits at a ratio of 1:1 co-assemble into 8-mer_Δ3C_, while 24 of the third subunits assemble into a 24-mer_Δ3C_ protein nanocage

To determine the possible difference in structure between 8-mer_Δ3C_ and NF-8, the crystal structure of 8-mer_Δ3C_ was also resolved. Similar to NF-8, 8-mer_Δ3C_ also comprises eight of H_α_- and H_β_-type subunits at a ratio of 1:1, which assemble into a bowl-like structure with an outer diameter of around 9 nm (Fig. 4a). The crystal structure of 8-mer_Δ3C_ is in good agreement with its structure in solution characterized by analytical ultracentrifugation and TEM (Figs. 2c, e). However, removal of the intra-subunit S–S bonds in NF-8 did not inhibit the formation of the H_α_-type subunit in 8-mer_Δ3C_, suggesting that the intra-subunit S–S bonds are not essential stabilizing forces for the structure of H_α_. Although the structure of 8-mer_Δ3C_ is similar to that of NF-8, their packing pattern in the crystal is completely different from each other. For example, the side view of the crystal structure revealed that 8-mer_Δ3C_ molecules array in a repeating side-to-side pattern to form two-dimensional (2D) protein layers (Fig. 4c), where two adjacent bowl-like 8-mer_Δ3C_ molecules having opposite orientations are connected by two salt bridges (Fig. 4d). The formed 2D layers further arrange in the vertical direction to create 3D porous protein assemblies (Fig. 4b). In contrast, NF-8 exhibits a different packing pattern in its crystal where six of NF-8 protein molecules assemble into a 48-mer protein cage^13^. It is worth noting that one polypeptide of mutant Δ3C is able to fold into three types of subunits: H_α_, H_β_, and H_γ_; subsequently, the first two kinds of subunits co-assemble into 8-mer_Δ3C_, while 24 of the third-type subunits self-assemble into 24-mer_Δ3C_ (Fig. 5).

### Design of a 16-mer nanocage from NF-8

**Fig. 6:**
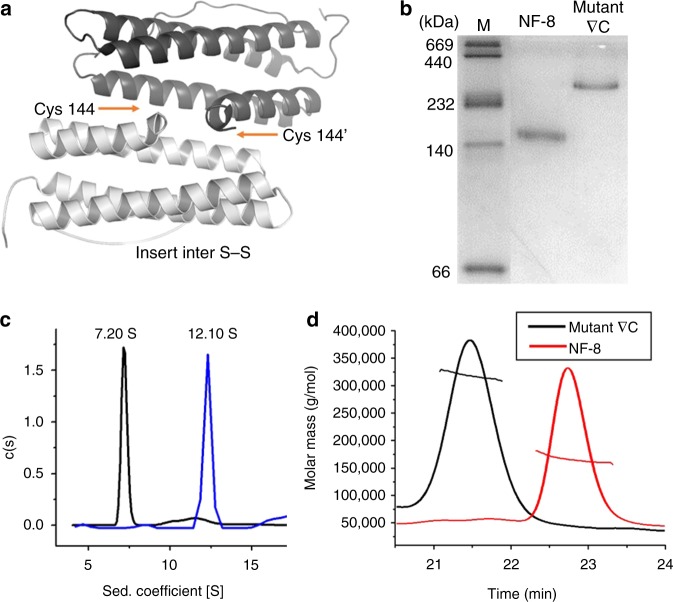
Preparation and characterization of mutant ∇C. **a** Structural design of mutant ∇C by insertion of Cys144 to the H_α_ subunit by genetic modification in order to produce an inter S–S bond. **b** Native PAGE analyses of mutant ∇C and NF-8. Source data are provided as a Source Data file. **c** Sedimentation coefficient distribution for mutant ∇C and NF-8. **d** SEC-MALS analyses of mutant ∇C with NF-8 as a control

The above results demonstrated that the intra-subunit S–S bond plays an important role in controlling protein tertiary and quaternary structure. We wonder whether inter-subunit S–S bonds can also be utilized as a linkage for the construction of a discrete protein architecture by using the same building block. To this end, we also chose NF-8 as a building block to create another protein species. Our approach is to insert an inter-subunit disulfide bond at the outer edge of each H_α_ subunit, which could bridge NF-8 molecules together to form a larger protein architecture. Upon inspection, we deemed the amino acid position 144 (originally aspartic acid) located at the middle of the D-helix of the H_α_ subunit (Supplementary Figure 8) to be well suited for constructing a motif for inter-subunit S–S interactions (Fig. 6a). The side chain at this position is protruded toward outside, and thus substitution of Asp144 by Cys could provide sufficient room for such inter-subunit S–S interactions between protein building blocks. Based on these considerations, we made a NF-8 mutant named ∇C, in which only Asp144 was mutated to Cys (Supplementary Figure 3). After *E. coli* cells expressing mutant ∇C were lysed, we found that there was only one overexpressed band appearing in native PAGE, which exhibited a different electrophoretic behavior from that of NF-8 (Fig. 6b), indicative of the formation of a larger protein. The yield of ∇C is around 30 mg per 1 L of culture medium.

**Fig. 7:**
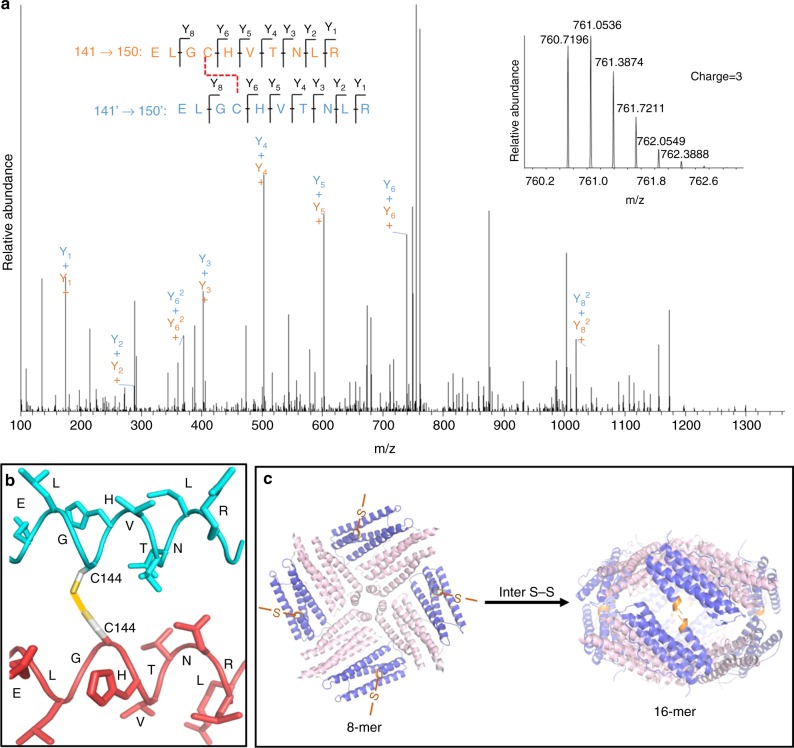
The formation of 16-mer_∇C_ by the inter S–S bond linkage. **a** Annotated MS/MS spectrum of the inter disulfide bond. The inset corresponds to the MS spectrum of peptides. **b** Structural analysis of the inter disulfide bond based on the MS/MS spectrum. **c** The possible mechanism of the formation of 16-mer_∇C_ with NF-8 as building blocks controlled by the inter S–S bond (Cys144–Cys144′)

To gain insight into the characteristics of mutant ∇C, we purified it to homogeneity (Fig. 6b). SDS-PAGE analyses revealed that this protein consists of one kind of subunit, the MW of which is about 20 kDa (Supplementary Figure 9, inset). The accurate MW of this mutant subunit was obtained as 20592 Da by MALDI–TOF–MS (Supplementary Figure 9), being in agreement with its theoretical value (20543 Da). The sedimentation coefficient of native mutant ∇C is ~12.10 S, a value being larger than that of NF-8 (s_20,w_ = 7.20 S). Size-exclusion chromatography combined with multi-angle light scattering (SEC-MALS) was performed to determine the MW of mutant ∇C in its native form. We found that this designed protein was eluted from a Superdex 200 10/300 GL (GE Healthcare) column in a single peak at a volume of about 21.5 mL (Fig. 6d), giving the weight-averaged molecular mass as 318 ± 10 kDa, which is ~2-fold larger than that of NF-8 (165 ± 6 kDa), demonstrating that it is a 16-mer protein assembly in solution, and therefore it is named as 16-mer_∇C_. Subsequently, we used TEM to visualize the morphology of 16-mer_∇C_ with NF-8 as a control sample. TEM results showed that 16-mer_∇C_ exhibits nearly the same morphology and size as NF-8 (Supplementary Figure 10a, b), suggesting that mutant ∇C could be a NF-8 dimer, namely two NF-8 building blocks polymerize in a face-to-face manner to form an oval-shaped 16-mer protein cage induced by the inter-subunit S–S bond. If this is the case, one would expect that iron cores can be formed within 16-mer_∇C_ because of its shell-like structure, whereas NF-8 cannot due to its open structure. As expected, TEM analyses showed that iron cores with 500 iron/protein shells can be successfully generated with 16-mer_∇C_ as a biotemplate according to our reported method^33^; however, such iron cores cannot be observed with NF-8 under the same experimental conditions (Supplementary Figure 10c, d); these findings approve the above conclusion that 16-mer_∇C_ has a shell-like structure. To clarify the disulfide connectivity for 16-mer_∇C_, MS/MS analysis was performed according to a reported method^34^. As expected, the inherent intra-subunit S–S bond was identified to form between Cys90 and Cys102 (Supplementary Figure 11). Additionally, it was found that an inter-subunit S–S bond formed between two Cys144 residues coming from two identical subunits, respectively (Fig. 7a, b), confirming our design. Dynamic light scattering (DLS) analyses showed that the 16-mer_∇C_ protein nanocage is stable over the pH range of 7.0–10.0 (Supplementary Figure 12). Taken together, it appears that the incorporation of a well-placed inter-subunit disulfide bond has the good potential to build a discrete protein architecture.

### The construction of a 48-mer nanocage from NF-8

**Fig. 8:**
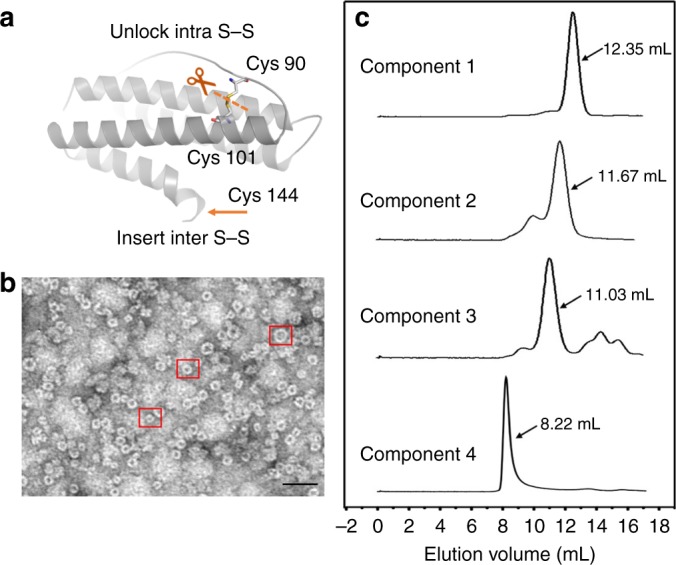
Deletion of intra S–S and insertion of inter S–S yields mutant Δ3C-∇C. **a** Redesign of the H_α_ subunit by deletion of intra S–S and insertion of inter S–S. **b** TEM image of overexpressed products related to the mutant Δ3C-∇C. Three typical nanocages were highlighted by a red cube. Scale bars represent 50 nm. **c** Four components related to the mutant Δ3C-∇C can be separated by size-exclusion chromatography

Either removal of the inherent intra-subunit S–S or addition of the extra inter-subunit S–S can facilitate the conversion of NF-8 into different protein species. This raises an interesting question as to what if we remove the intra-subunit S–S of NF-8 while inserting an extra S–S at the outside of NF-8 (Fig. 8a). To answer this question, we prepared the third NF-8 mutant termed Δ3C-∇C where cysteine residues (Cys90, Cys102, and Cys130) were replaced by alanine (Ala) while Asp144 was mutated to Cys (Supplementary Figure 3). After *E. coli* cells expressing this mutant were lysed, we found that four overexpressed products occurred as shown in native PAGE (Supplementary Figure 13a), suggesting that one polypeptide can simultaneously produce four different protein species. The total yield of these four species is about 45 mg per 1 L of culture medium under the present conditions. Three of them exhibited the nearly same electrophoretic behavior as NF-8 (band 1), 16-mer_∇C_ (band 2), and 24-mer_Δ3C_ (band 3), respectively, suggesting that they could have similar protein assemblies. After preliminary purification, we used TEM to visualize the morphology of the mixture, and found the largest protein assembly, the exterior diameter of which is about 17 nm (Fig. 8b). To confirm this observation, analytical ultracentrifugation analyses were carried out, likewise showing that there are four protein species in solution related to the mutant Δ3C-∇C. The first three peaks correspond to 8-mer, 16-mer, and 24-mer, respectively, based on their sedimentation coefficients, while the fourth peak represents a different kind of protein assembly with the largest sedimentation coefficient of 22.50 S in solution (Supplementary Figure 13b).

To obtain their structural information, this mixture was further purified by using size-exclusion chromatography, and eventually four protein components could be separated (Fig. 8c). Subsequently, we used these four protein assemblies to screen for their suitable crystallization conditions, respectively. However, we only found conditions which are suitable for the growth of crystals with the 24-mer_Δ3C-∇C_ and the above largest species, but not for 8-mer_Δ3C-∇C_ and 16-mer_Δ3C-∇C_ crystals. We first solved the crystal structure of 24-mer_Δ3C-∇C_ (Supplementary Figure 14 and Supplementary Table 2). As expected, this protein consists of 24 H_γ_-type subunits, the crystal structure of which is nearly the same as that of 24-mer_Δ3C_, confirming the above observation by native PAGE and analytical ultracentrifugation (Supplementary Figure 13a, b).

For the above-mentioned largest protein species, we found that two conditions are suitable for the growth of crystals, namely one condition having no Mg^2+^ and another condition containing Mg^2+^. Subsequently, these two conditions were further optimized in a manual plate setup with hanging-drop vapor diffusion to increase crystal size and quality. Under the crystallization condition without Mg^2+^, we eventually obtained large single crystals suitable for X-ray diffraction studies. We solved the crystal structures at a high resolution of 2.699 Å (Supplementary Figure 15, Supplementary Tables 1 and 2), and found that this large protein species is a heteropolymer which consists of 48 subunits of H_α_ and H_β_ at a ratio of 1:1, and thus it is named as 48-mer_Δ3C-∇C_. The exterior and interior diameter of this 48-mer is about 17 nm and 13 nm in crystals, respectively. Consistent with this observation, TEM analyses showed that the outer diameter of the 48-mer is also ~17 nm (Supplementary Figure 16). Thus, controlling the disulfide bond in protein building blocks can facilitate the conversion of NF-8 into not only the 16-mer and 24-mer protein architectures, but also an even larger 48-mer protein nanocage. It is worth noting that, similar to the mutant Δ3C, one polypeptide of the mutant Δ3C-∇C likewise forms H_α,_ H_β_, and H_γ_ subunits, and these three subunits can simultaneously stay in one solution and assemble into 24-mer and 48-mer protein nanocages, these findings being in accordance with the above observation with the mutant Δ3C (Fig. 5).

**Fig. 9:**
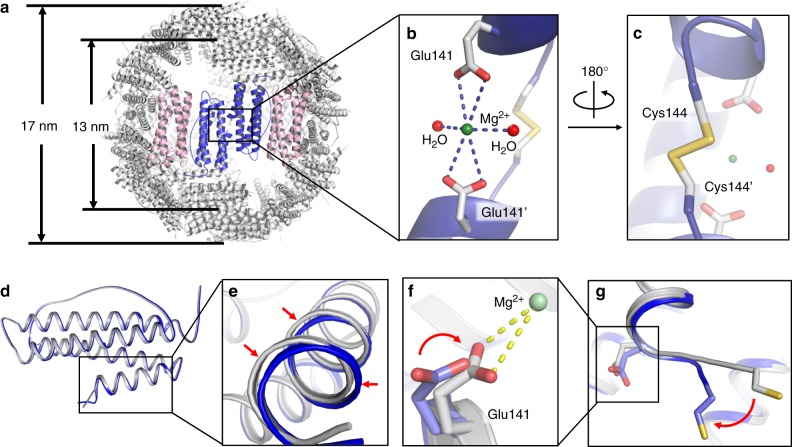
The crystal structure of 48-mer_Δ3C-∇C_ in the presence of Mg^2+^. **a** The crystal structure showed that a 48-mer_Δ3C-∇C_ molecule is composed of two types of subunits, H_α_ (blue) and H_β_ (pink). The exterior and inner diameter of a 48-mer_Δ3C-∇C_ nanocage is 17 nm and 13 nm, respectively. **b** Close-up view of Mg^2+^ coordination with Glu141 and Glu141′ from two H_α_ subunits and two H_2_O molecules. **c** On the opposite side, Cys144 and Cys144′ from such two H_α_ subunits formed an inter disulfide bond. **d** Superposition of two H_α_ subunits of two 48-mer protein nanocages corresponding to the mutant Δ3C-∇C in the presence (gray) and absence (blue) of Mg^2+^. **e** The D α-helix moved for about 0.9 Å after Mg^2+^ coordination with Glu141. **f** A change in Glu141 conformation upon its coordination with Mg^2+^. **g** An obvious movement of Cys144 was induced by Mg^2+^ coordination

Further crystal analyses revealed that no inter-subunit S–S bond was formed in the 48-mer_Δ3C-∇C_ nanocage. Agreeing with this finding, TEM analyses showed that, upon dissolving the crystals in buffer, 48-mer_Δ3C-∇C_ molecules were degraded into small species after 24 h, the size of which is identical to that of NF-8, and then such small species were associated with each other (Supplementary Figure 17a). These results suggested that 48-mer_Δ3C-∇C_ is unstable in solution, and it is constructed directly by using the 8-mer as building blocks. However, we excitedly noted that the inter-subunit S–S bond formed at subunit–subunit interfaces of the 48-mer protein nanocage when its crystals grew under the crystallization conditions containing Mg^2+^. The structure of the 48-mer protein nanocage in the presence of Mg^2+^ exhibited nearly the same geometry as the above-mentioned 48-mer with exterior and inner diameter of 17 nm and 13 nm, respectively (Fig. 9a). Except for the inter-subunit S–S bond, one magnesium ion is bound to two acidic residues (Glu141 and Glu141′) and two water molecules by coordination bonds at the same subunit–subunit interfaces (Fig. 9b). Comparative analyses indicated that the formation of Mg^2+^ coordination bonds is a prerequisite for the generation of the inter-subunit S–S bonds in the 48-mer (Fig. 9c). Why is Mg^2+^ coordination so important for the formation of the inter-subunit S–S? The answer to this question may lie in the difference in crystal structure between these two 48-mer protein nanocages in the presence and absence of Mg^2+^. The formation of Mg^2+^coordination bonds with Glu141 causes a movement of the D-helix of two H_α_ subunits by 0.9 Å; consequently, Cys144 and Cys144′ residues from two H_α_ subunits are in close proximity, resulting in the generation of the inter-subunit S–S bond (Figs. 9d–g). Consistent with the existence of the inter-subunit S–S bonds in the protein crystal structure, we found that this large protein cage is stable in solution based on the fact that its size and shape kept unchanged over the time range of 24 h (Supplementary Figure 17b and 17c) after the crystals were dissolved in buffer. Thus, the cooperation of the inter-subunit S–S and metal coordination bonds located at subunit–subunit interfaces (Supplementary Figure 18) greatly improved the stability of the 48-mer protein nanocage. However, we found that protein association occurs to some extent with the 48-mer protein nanocage at 7.0 as suggested by DLS (Supplementary Figure 19). This phenomenon is most likely caused by the larger outer surface area (~900 nm^2^) of the 17-nm-diameter nanocage, which would greatly increase the intermolecular interactions. In contrast, at pH 3.0, protein nanocage disassembly occurs, resulting in the formation of subunits (Supplementary Figure 19). These results suggested that the pH stability of the 48-mer protein nanocage in solution is lower than that of other protein architectures (8-mer, 16-mer, and 24-mer). Furthermore, stopped-flow UV-visible results showed that the rate of iron oxidation catalyzed by the 48-mer is similar to that of native HuHF at 8 Fe^2+^/subunit (Supplementary Figure 19c), suggesting that such large assembly hardly affects the original ferroxidase activity.

It has been known that ordered assembly of nanoscale building blocks depends on the assembly conditions. We rationalized that the crystallization setup used in protein crystallography could be applicable to the study of the conversion of NF-8 into other protein architectures in solution. These considerations combined with the fact that the stability of the 48-mer nanocage was greatly improved by the presence of magnesium ions raise the possibility that treatment of *E. coli* (which expressed the mutant Δ3C-∇C) with magnesium salts could facilitate the conversion of NF-8 into the 48-mer. To test this hypothesis, we conducted another experiment where extra magnesium salts were added to the medium for the culture of *E. coli*. After cells were lysed, native PAGE analyses showed that the amount of overexpressed 48-mer protein was pronouncedly increased, while the 16-mer protein and 8-mer protein architectures were expressed to a much less level (Supplementary Figure 20a). Differently, the 24-mer overexpressed level was almost unchanged in both the presence and absence of magnesium salts. Support for this view comes from TEM results showing that only two kinds of protein nanocages occurred in solution, namely the 24-mer and the 48-mer protein nanocages (Supplementary Figure 20b). These findings suggested that all of the 8-mer, 16-mer, and 48-mer are composed of H_α_- and H_β_-type subunits, and thus they can interconvert with each other at the level of the protein quaternary structure depending on experimental conditions.

## Discussion

While the conversion of protein assemblies into symmetrical analogs with lower order by targeted disruption of noncovalent interactions at subunit interfaces has a long track record of success^35–39^, the conversion of low-order symmetrical protein assembly into its analog with higher symmetry is a challenge. We have described here a protein-engineering approach to convert the 8-mer protein assembly with *C*_*4*_ symmetry into the 16-mer, 24-mer, and 48-mer protein nanocages with higher symmetry by controlling the intra- or inter-subunit disulfide bond. More interestingly, the fabricated protein nanocages (16-mer, 24-mer, and 48-mer) is composed of three different types of subunits (H_α_, H_β_, and H_γ_) which are derived from one polypeptide; H_α_ and H_β_ subunits are responsible for the formation of the 16-mer and 48-mer, while the H_γ_ subunit corresponds to the generation of the 24-mer. This approach has allowed us to address the significance of both intra- and inter-subunit disulfide bonds in protein assembly and to gain specific insights into either the formation of subunit or subunit interactions that direct protein nanocage assembly.

The mechanism of the conversion of the 8-mer into three different protein nanocages can be divided into two categories. The first one is referred to as a “subunit refolding” mechanism which takes place at the level of protein tertiary structure mediated by intra-subunit S–S bonds. The conversion of NF-8 into 24-mer_Δ3C_ belongs to the first. Based on the crystal structure, it is clear that the initial H_α_ and H_β_ subunits of NF-8 are converted into the H_γ_ subunit in 24-mer_Δ3C_ upon the deletion of the intra-subunit S–S (Fig. 3). We believe that the conversion of NF-8 into 24-mer_Δ3C_ is most likely derived from the contribution of the intra-subunit S–S bond to the stability of the protein architecture. Support for this idea comes from the observation that the melting point (*T*_*m*_) of the 8-mer bowl-like NF-8 protein is about 76 °C, while its analog 8-mer_Δ3C_’s *T*_*m*_ decreases to 73 °C due to a dearth of the intra-subunit S–S bond (Supplementary Figure 21). The difference in *T*_*m*_ reflects the contribution of the intra-subunit S–S bond to the protein stability. Interestingly, we found that the *T*_*m*_ of 24-mer_Δ3C_ (83 °C) is higher than that of NF-8, which is an important reason why NF-8 can convert into 24-mer_Δ3C_ through the above-mentioned subunit refolding mechanism.

Differently, the fact that both NF-8 and 48-mer protein nanocages consist of H_α_ and H_β_ subunits (Fig. 9) suggests that NF-8 can serve as building blocks to directly construct the 48-mer protein nanocage at the level of the quaternary structure through the inter-subunit S–S linkage at protein interfaces. This corresponds to the second conversion mechanism. It has to be mentioned that the presence of Mg^2+^ during the construction of the 48-mer not only leads to the formation of metal coordination bond with amino acid residues, but also facilitates the generation of the inter-subunit S–S bond between Cys144 and Cys144′ (Fig. 9). These two different types of chemical bonds are cooperative to stabilize the structure of the 48-mer protein nanocage. Agreeing with this view, the above-mentioned 48-mer protein nanocage did not generate when the 8-mer protein architecture (NF-8) was incubated with Mg^2+^ in vitro at different pH values (6.0, 7.0, and 9.0) (Supplementary Figure 22). It is not surprising because NF-8 is not able to form the inter-subunit S–S bond due to a dearth of Cys144 (Supplementary Figure 3). These findings emphasize the importance of the cooperation of both the metal coordination and the inter-subunit S–S bonds for the construction of the stable 48-mer protein nanocage. The assembling manner of the 48-mer_Δ3C-∇C_ protein nanocage controlled by Mg^2+^ is reminiscent of the formation of ferritin from *Archaeoglobus fulgidus*. In the absence of ferrous ion, this specific ferritin occurs in solution as dimeric species, while these dimeric species self-assemble into 24-meric cage-like structures induced by addition of ferrous ions^40–42^.

Here, the occurrence of assemblies (16-mer, 24-mer, and 48-mer) with the 8-mer as building blocks recalls among natural proteins the case of clathrin coats. It has been known that clathrin can assemble into several regular assemblies, such as 78-mer, 108-mer, and 180-mer^43^. Thus, our reported construction of three different protein nanocages (16-mer, 24-mer, and 48-mer) from the 8-mer as building blocks provides a model to study the mechanism of natural protein architectures. The construction of different protein nanocages from NF-8 also reminds us of the formation of viral capsids. In the structures of the viral capsids, the pentamer is the common building block that can be used to build a variety of assemblies with different symmetries^7^.

Protein nanocages hold the great promise of ease of functionalization, intrinsic biocompatibility, and versatile platforms for encapsulation and delivery of a wide variety of non-physiological cargo molecules. Such properties have been difficult to reach with other protein or biomolecule assemblies. Therefore, it is of crucial importance to establish methods to construct such protein assemblies. Our results established a simple, effective method by which protein nanocages with different symmetries can be created from one kind of protein building block by disulfide-mediated conversion. It is worth noting that both intra- and inter-subunit disulfides can be employed to build protein nanocages. The utilization of the disulfide bond to control protein assembly is attractive from the structural perspective at two different levels: whereas the directionality and strength in the intra-subunit disulfide bond formed within the subunit can affect the geometry of the subunit structure and thereby controlling the formation of protein architecture, the inter-subunit disulfide bond formed at subunit–subunit interfaces offers the ability to construct multi-subunit protein assemblies at the level of the quaternary structure. The combination of the metal coordination bond and the inter-subunit S–S bond provides an alternative approach to improve the stability of the constructed protein nanocages. Compared with the reported strategy for the preparation of protein nanocages that usually requires intensive re-engineering of protein interfaces, the present construction approach that focuses on the conversion between different protein architectures by the disulfide bond motif is conceptually and operationally simple, which could bypass the immense challenge of controlling the noncovalent interactions that hold protein assemblies together.

## Methods

### Protein preparation

The coding sequence of NF-8 was synthesized and cloned into the pET-3a plasmid (Novagen). Mutagenesis of NF-8 was performed with the fast site-directed mutagenesis kit (TIANGEN Biotech Co., Ltd.). The primers used in this work are listed in Supplementary Table 3. After the transfection of plasmid into the *E. coli* strain BL21 (DE3), cells were grown at 37 °C with further induction by 200 μM isopropyl β-D-1-thiogalactopyranoside at 20 or 37 °C. The cells were collected by centrifugation after induction and resuspended in 50 mM Tris-HCl (pH 8.0) with a concentration of bacteria as 40 g/L. Subsequently, ultrasonication was used to disrupt the cells. The resulting protein was enriched from the supernatant by fractionation of ammonium sulfate, followed by dialysis against 50 mM Tris-HCl (pH 8.0). Then crude protein was subjected to an ion-exchange column, followed by gradient elution with 0–0.5 M NaCl. After further purification by a gel filtration column (Superdex 200, GE Healthcare), equilibrated with 50 mM Tris-HCl and 150 mM NaCl (pH 8.0), the resultant protein was used for the following experiments.

### Transmission electron microscopy (TEM) analyses

TEM experiments were performed as below: 10 μL of protein was applied to a carbon-coated copper grid. After excess solution removed with filter paper, the samples were negatively stained for 2 min with 2% uranyl acetate. TEM micrographs were imaged at 80 kV through a Hitachi H-7650 scanning electron microscope.

### Analytical ultracentrifugation sedimentation analyses

The experiments were performed at 10 ℃ in an XL-I analytical ultracentrifuge (Beckman–Coulter) equipped with Rayleigh Interference detection (655 nm). Protein samples (110 μl) were centrifuged at 50,000 r.p.m. for 8 h. All samples were prepared in buffer (50 mM Tris, pH 7.5). Interference profiles were collected every 6 min. Data analysis was conducted with the software Sedfit 11.7, GUSSI, and SEDPHAT (monomer–dimer model).

### SEC-MALS analysis

SEC-MALS experiments for mutant ∇C and NF-8 were performed using a DAWN-HELEOS II detector (Wyatt Technologies) coupled to a Superdex 200 column (GE Healthcare) in buffer (50 mM Tris, 150 mM NaCl, pH = 8.0) with a flow rate of 0.4 mL/min. Mutant ∇C and NF-8 (~1.0 mg/mL) was injected and data were analyzed using ASTRA 6 software (Wyatt Technologies) to determine the weight-averaged molecular mass.

### LC–MS/MS spectrum

Gel bands of proteins were cut for in-gel digestion, followed by mass spectrometry analyses^38^. Sequencing grade-modified trypsin was used in gel digestion at 37 ℃ overnight. The peptides were extracted twice with 50% acetonitrile aqueous solution containing 1% trifluoroacetic acid for 1 h. Then the further concentrated peptides were separated with a Thermo-Dionex Ultimate 3000 HPLC system, which was directly interfaced with a Thermo Orbitrap Fusion Lumos mass spectrometer. Mobile phase A consisted of 0.1% formic acid, and mobile phase B was added with 100% acetonitrile. An LTQ-Orbitrap mass spectrometer was operated in a data-dependent acquisition mode using Xcalibur 4.1 software. MS/MS spectra from each LC–MS/MS run were searched against the ferritin sequence using Byonic^TM^ Version 2.8.2 (Protein Metrics) searching algorithm.

### Dynamic light scattering (DLS)

DLS experiments were performed at 25 °C using a Viscotek model 802 dynamic light scattering instrument (Viscotek, Europe). The OmniSIZE 2.0 software was used to calculate the size distribution of samples. For all samples, protein concentration was 1.0 μM, and proteins were buffered in 20 mM Tris-HCl, pH 8.0 with different concentrations of NaCl.

### Differential scanning calorimetry (DSC)

A differential scanning calorimeter (Nano-DSC, TA Instruments) was used for measurement with the following settings: temperature was set from 30 to 100 °C with an increasing rate at 1.0 °C/min. A result of control buffer was used to subtract the baseline for melting temperature (*T*_*m*_) calculation by software. Precision data of each protein were calculated by repeated scans in duplicate.

### Crystallization, data collection, and structure determination

Purified mutants were buffered in 10 mM Tris-HCl (pH 8.0) after three-times dialysis, and were then concentrated to 10 mg/mL. Their crystals were obtained by the hanging-drop vapor diffusion method at different conditions, which were shown in Supplementary Table 1. X-ray diffraction data were collected at Shanghai Synchrotron Radiation Facility (SSRF) (BL17U and BL19U) with merging and scaling by HKL-3000 software. Data-processing statistics are displayed in Supplementary Table 2. The structures were determined by molecular replacement using coordinates of human H ferritin and NF-8 (PDB code 2FHA and 5GN8) as the initial model using the MOLREP program in the CCP4 program. Following refinement and manual rebuilding were carried out by PHENIX and COOT, respectively. All figures of the resulting structures were produced using PyMOL.

### Reporting summary

Further information on experimental design is available in the Nature Research Reporting Summary linked to this article.

## Supplementary information

Supplementary Information

Reporting Summary

Source Data

## Data Availability

Coordinates and structure factors are deposited in the Protein Data Bank under the accession PDB IDs: 6IPQ (24-mer_Δ3C_), 6IPC (8-mer_Δ3C_), 6J7G (24-mer_Δ3C-∇C_), 6IPP (48-mer_Δ3C-∇C①_), and 6IPO (48-mer_Δ3C-∇C②_). Other data are available from the corresponding authors upon reasonable request. The source data underlying Figs. 2b and 6b and Supplementary Figs. 4, 9, 13a, 17c and 20a are provided as a Source Data file.
