# Supplementary material for: Disulfide-mediated conversion of 8-mer bowl-like protein architecture into three different nanocages
Source: Nat Commun. 2019 Feb 15;10:778. doi: 10.1038/s41467-019-08788-9 (PMC6377661; doi:10.1038/s41467-019-08788-9)
Supplement: Supplementary file 1 — Supplementary Information [file 41467_2019_8788_MOESM1_ESM.pdf]

Supplementary Information

Disulfide-mediated conversion of 8-mer bowl-like protein architecture into three different nanocages

Zang et al.

Supplementary Figures

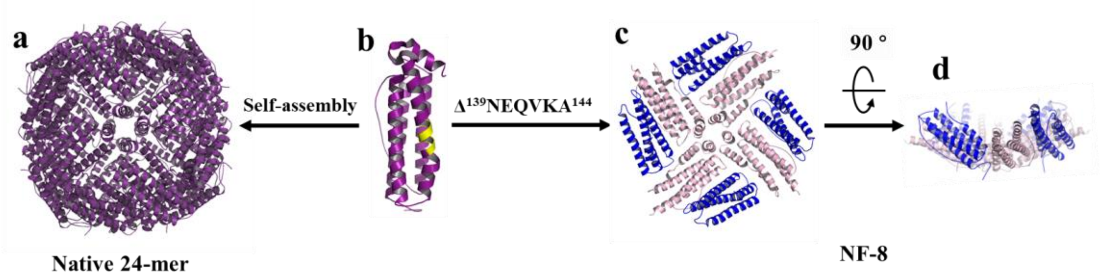

Supplementary Figure 1. The construction of a bowl-like protein NF-8 from HuHF. (**a**) Crystal structure of a tetraeicosameric recombinant HuHF (PDB ID: 2FHA), which is composed of 24 subunits. (**b**) Structure of HuHF subunit. Deletion of six amino acid residues (which have been highlighted in yellow) at D-helix located at the C_3-C_4 interface of native HuHF resulted in its complete conversion into a bowl-like 8-mer protein architecture (NF-8) in solution. (**c**) Top view of NF-8. (**d**) Side view of NF-8 which has a bowl-like structure.

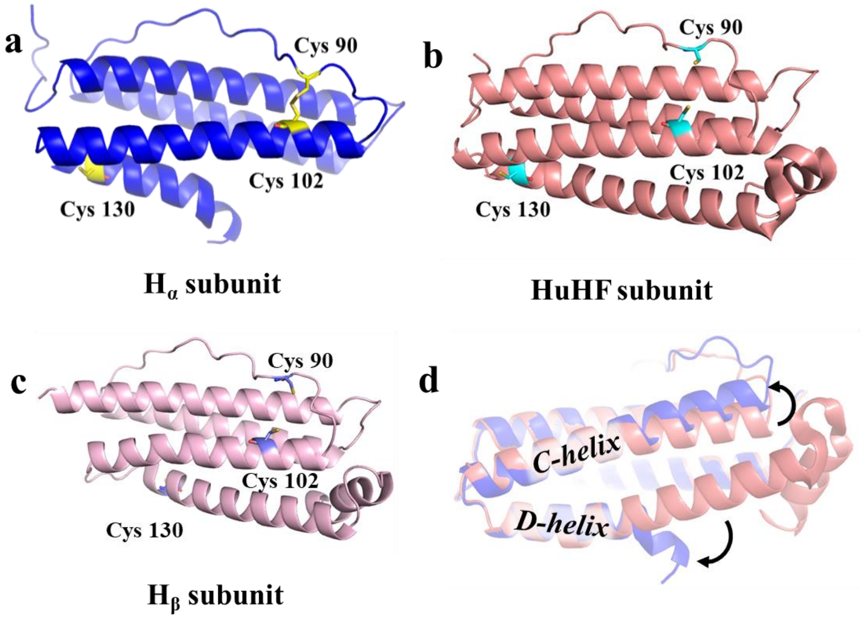

Supplementary Figure 2. The crystal structure of HuHF and NF-8 subunits. (**a**) NF-8 is composed of 4 H_α and 4 H_β subunits. Each H_α subunit consists of one intra-subunit S-S bond (yellow color) formed between Cys90 and Cys102. Cys130 is a single free cysteine which is also highlighted in yellow. (**b**) Each native ferritin subunit lacks such intra-subunit S-S bond and consists of 3 free cysteines. (**c**) H_β subunit is also devoid of such intra-subunit S-S bond, and contains 3 free cysteines. (**d**) Superposition of native H subunit (orange) and H_α subunit (blue) revealed that the formation of the intra-subunit S-S bond resulted in movement of both D α-helix and C α-helix towards different directions.

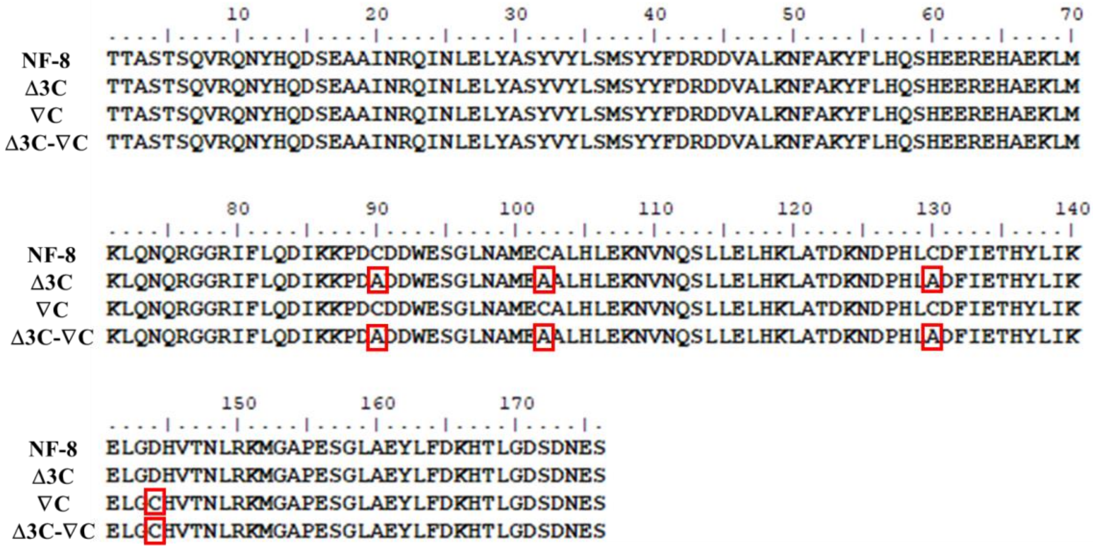

Supplementary Figure 3. Complete amino acid sequences of NF-8 and its three mutants. Mutation sites for all three mutants have been highlighted in red box.

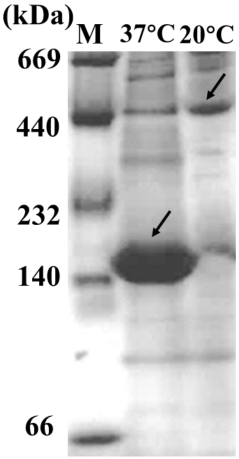

Supplementary Figure 4. Native PAGE analyses of mutant Δ3C. The main products were overexpressed at different temperatures. Source data are provided as a Source Data file.

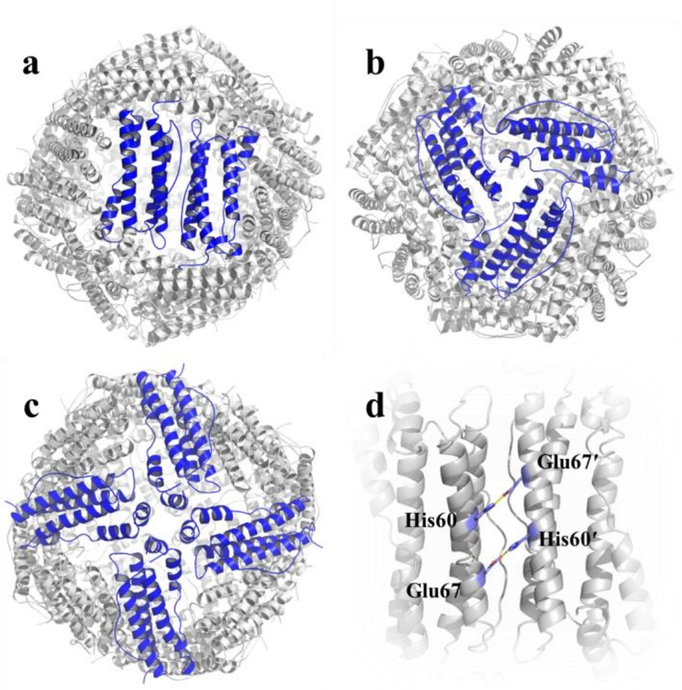

Supplementary Figure 5. Crystal structure of 24-mer$_{\Delta 3C}$. View of 24-mer$_{\Delta 3C}$ crystal structure down the two fold (**a**), three-fold (**b**) and four-fold symmetry axes (**c**). The 24-mer$_{\Delta 3C}$ protein nanocage is a homopolymer which comprises 24 H_{γ} subunits. (**d**) The key contacts at C_2 interface of 24-mer$_{\Delta 3C}$ where two salt bridges are formed by His60 and Glu67, representing major interactions.

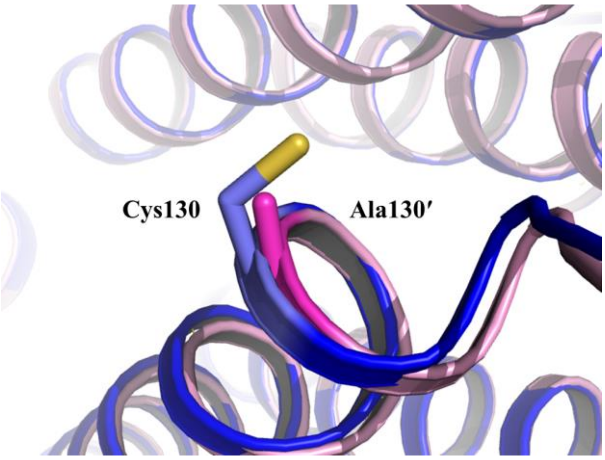

Supplementary Figure 6. Structural comparison of NF-8 and 24-mer$_{\Delta 3C}$. . NF-8 and 24-mer$_{\Delta 3C}$ subunits were shown in blue and pink, respectively. Cys130 and Ala130' were highlighted.

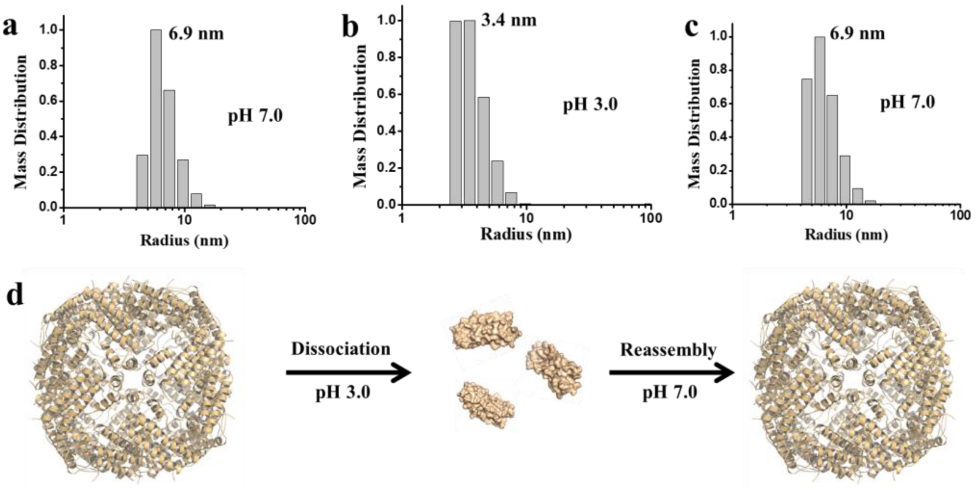

Supplementary Figure 7. Disassembly and reassembly of 24-mer$_{\Delta 3C}$ nanocage by pH. (**a**) Dynamic light scattering (DLS) analyses of 24-mer$_{\Delta 3C}$ nanocage at pH 7.0. There is one population with the hydrodynamic radius (R_H) of 6.9 nm in solution. (**b**) At 3.0, the R_H of protein species in solution decreased to 3.4 nm due to protein nanocage disassembly into subunits. (**c**) When adjusting pH back to pH 7.0, the R_H of protein species in solution increased back to 6.9 nm due to subunit reassembly into protein nanocage. (**d**) Schematic description of disassembly and reassembly of the newly prepared 24-mer ferritin-like nanocage, 24-mer$_{\Delta 3C}$, controlled by pH.

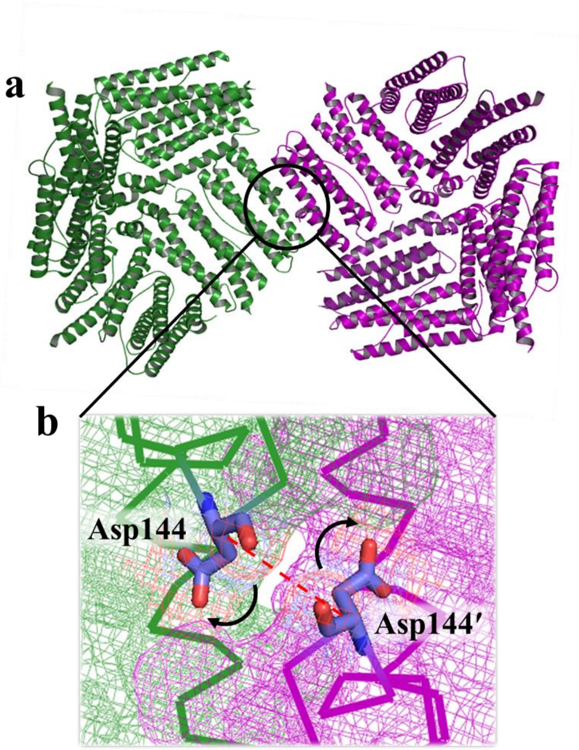

Supplementary Figure 8. Packing pattern of two NF-8 molecules in crystal. (**a**) H_α subunits are directly involved in intermolecular interactions of two NF-8 molecules in crystal[13]. (**b**) There are electrostatic repulsive interactions from acidic residues Asp144 and Asp144' which are contributed from two H_α subunits[13].

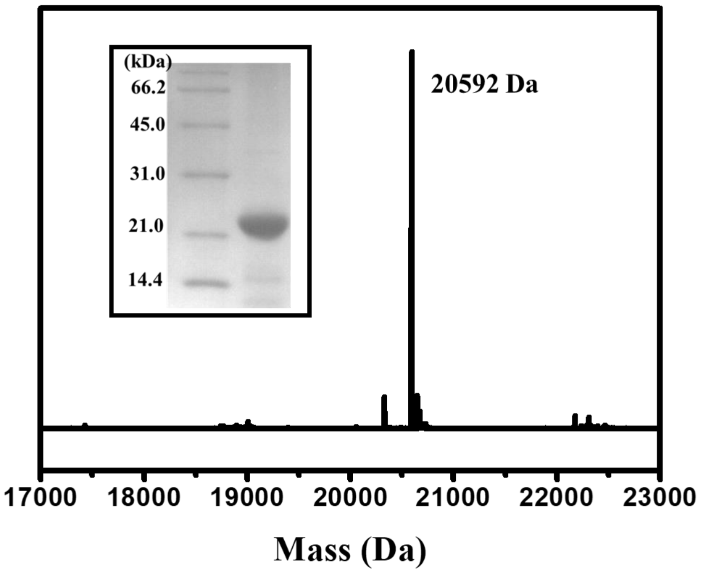

Supplementary Figure 9. The determination for the MW of mutant ∇C subunit. MALDI-TOF-MS results indicated the MW of ∇C as 20591 Da. SDS-PAGE analysis of mutant ∇C was shown in the inset. Source data are provided as a Source Data file.

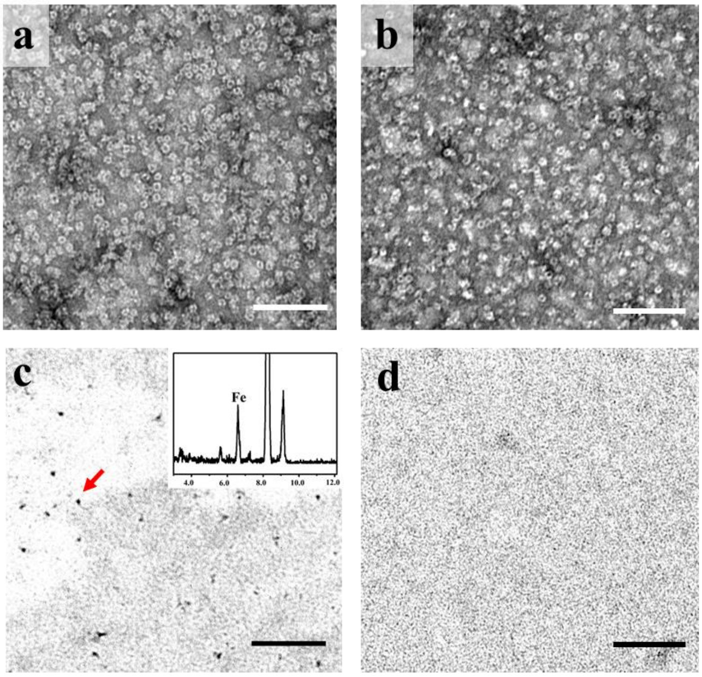

Supplementary Figure 10. TEM images of mutant ∇C and NF-8. TEM images of mutant (**a**) ∇C and (**b**) NF-8 upon stained by uranium. Scale bars represent 50 nm. (**c**) TEM images of iron cores formed when mutant ∇C was used as a biotemplate. Energy-dispersive X-ray (EDX) analysis confirmed the presence of iron (inset). (**d**) No iron core was formed with NF-8 under the same experimental conditions as mutant ∇C. No uranium staining was used. Scale bars represent 50 nm.

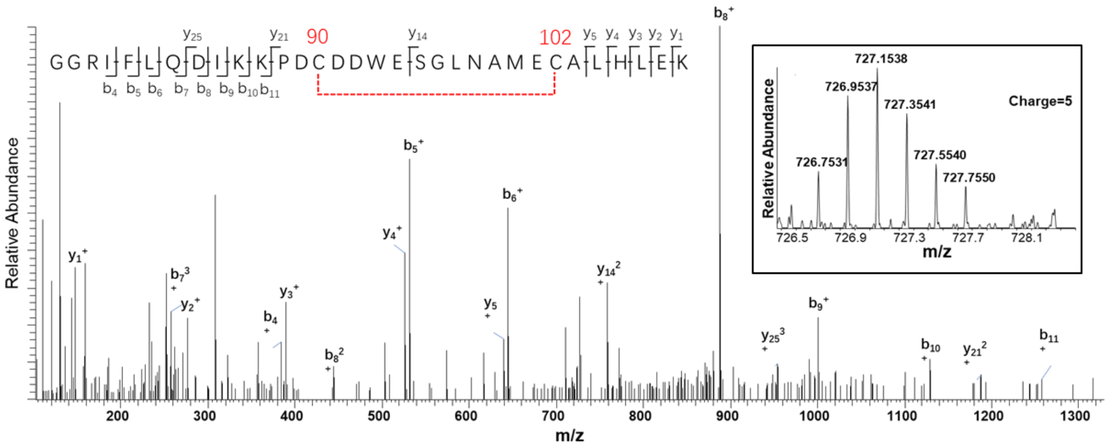

Supplementary Figure 11. MS/MS spectrum of the intra-subunit S-S. Inset corresponds to the MS spectrum of peptides.

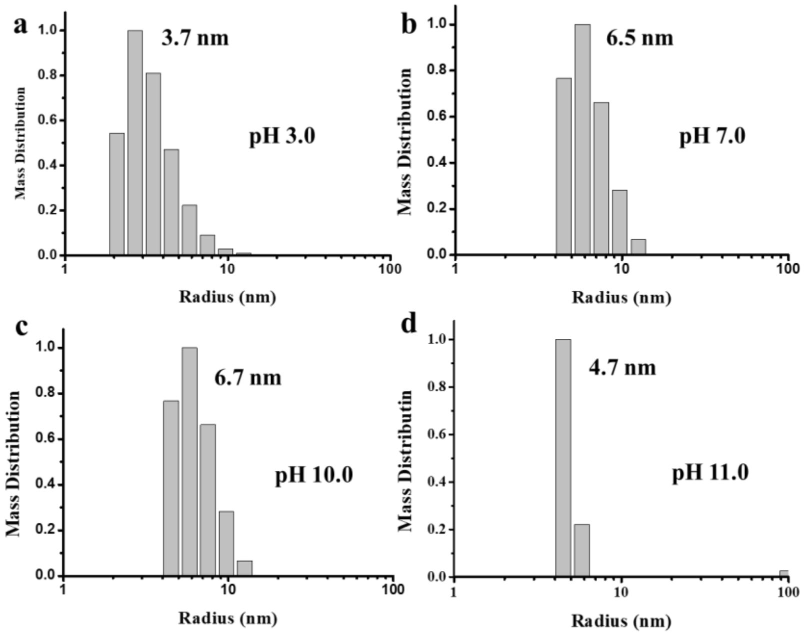

Supplementary Figure 12. Mass distribution of different particle sizes of 16-mer$_{VC}$. The primary species of 16-mer$_{VC}$ in solution over the pH rang of 7.0 to 10.0 has a hydrodynamic radius (R_H) of 6.5-6.7 nm (**b, c**), corresponding to protein monomer, suggesting that this protein nanocage is stable during this pH range. In contrast, at pH 3.0 or 11.0, the R_H decreases to 3.7 or 4.7 nm (**a, d**), suggesting that protein disassembly occurs.

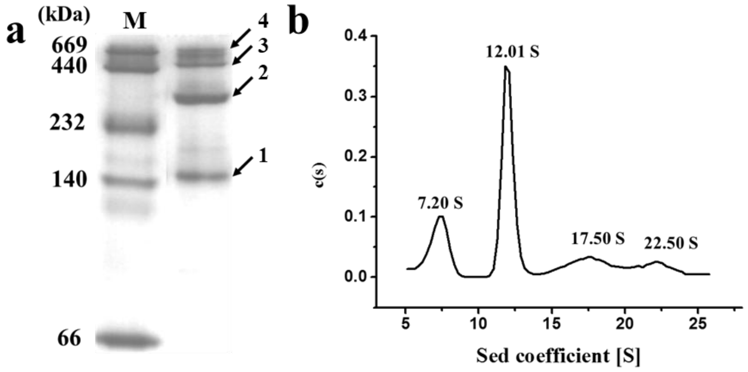

Supplementary Figure 13. Characterization of mutant Δ3C-∇C. (**a**) Native PAGE analyses of mutant Δ3C-∇C revealed four overexpressed products, three of which were identified to be 24-mer (band 3), 16-mer (band 2), and 8-mer (band 1) assemblies based on their electrophoretic behavior, respectively. Band 4 represents the new protein species which has the largest MW. Source data are provided as a Source Data file. (**b**) Sedimentation coefficient distribution of four overexpressed products coming from mutant Δ3C-∇C.

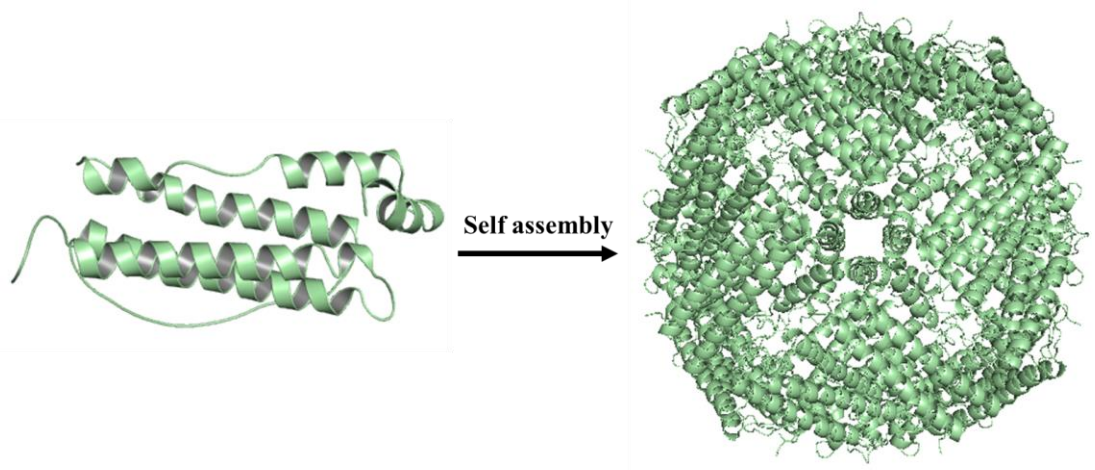

Supplementary Figure 14. The crystal structure of 24-mer$_{\Delta 3C\text{-}\nabla C}$. This new protein nanocage is a homopolymer which is composed of 24 identical H_{γ} subunits.

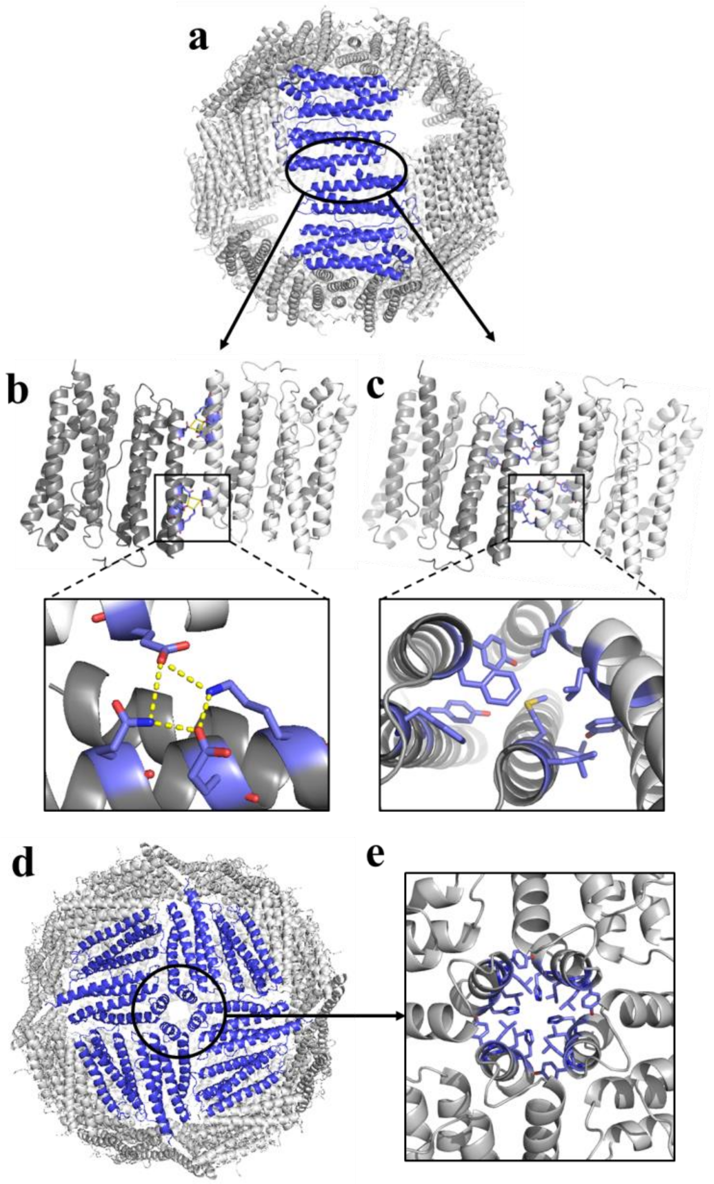

Supplementary Figure 15. The *C_2* interface of 48-mer$_{\Delta 3C\text{-}\nabla C}$ nanocage (**a**) Protein viewed down the C_2 symmetry axis. The noncovalent interactions at the C_2 interface play an important role in maintaining the structure of 48-mer$_{\Delta 3C\text{-}\nabla C}$ nanocage. (**b**) The salt bridges are formed at the two surface-ends of the C_2 interface. (**c**) The hydrophobic interactions at the two ends of the C_2 interface. (**d**) The C_4 interface of 48-mer$_{\Delta 3C\text{-}\nabla C}$ nanocage. (**e**) The main hydrophobic interactions at the C_4 interface are also responsible for protein assembly.

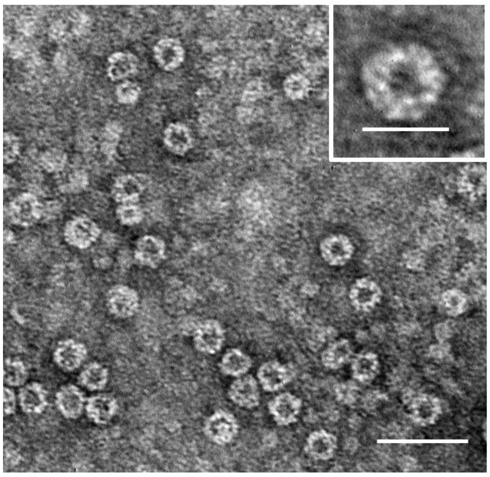

Supplementary Figure 16. TEM image of the 48-mer$_{\Delta 3C\text{-}\nabla C}$ nanocage. Scale bars represent 50 nm. Inset is the enlarged picture of one nanocage with the exterior diameter as about 17 nm.

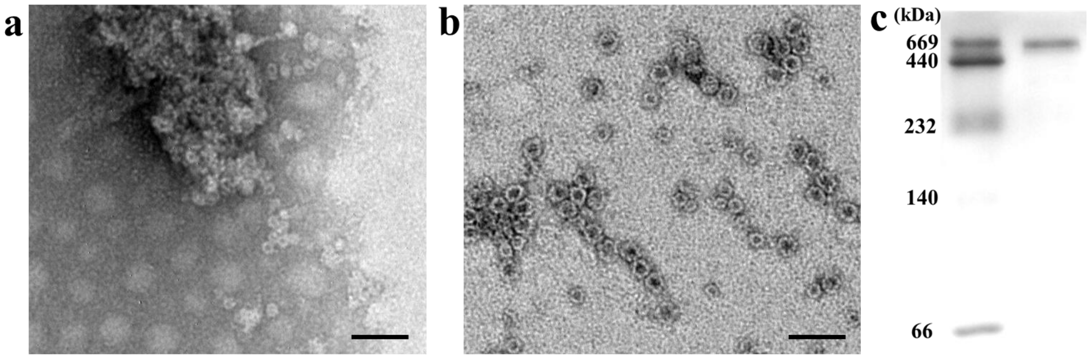

Supplementary Figure 17. The stability analyses of 48-mer$_{\Delta 3C\text{-}\nabla C}$ protein nanocage. (**a**) In the absence of Mg^{2+}, the 48-mer protein nanocages are unstable in solution, and are degraded into 8-mer protein architectures after 24 h. The formed 8-mer molecules were associated with each other. (**b**) The morphology of the 48-mer protein nanocages almost keep unchanged in solution in the presence of Mg^{2+} after 24 h. Scale bars represent 50 nm. (**c**) Native PAGE of the 48-mer protein in the presence of Mg^{2+}. Source data are provided as a Source Data file.

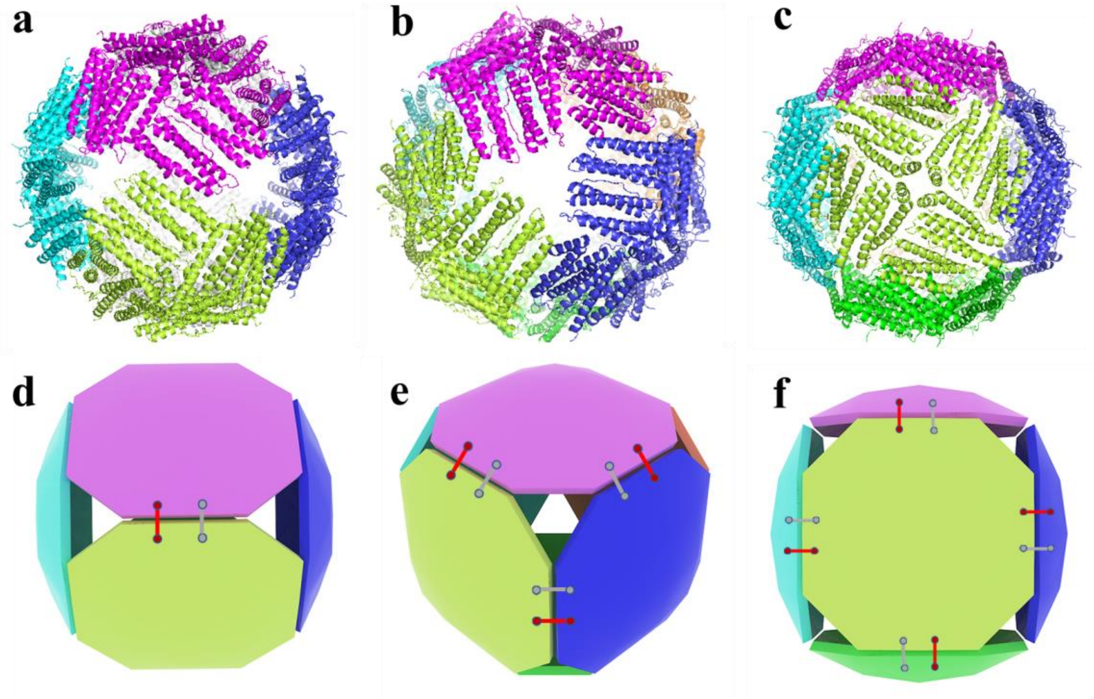

Supplementary Figure 18. Crystal structure of 48-mer$_{\Delta 3C\text{-}\nabla C}$. View of the crystal structure of 48-mer$_{\Delta 3C\text{-}\nabla C}$ down the two-fold symmetry (**a**), three-fold symmetry (**b**) and four-fold symmetry axes (**c**). Modeling description of 48-mer$_{\Delta 3C\text{-}\nabla C}$ stabilized by the inter-subunit S-S (grey) and Mg^{2+} coordination bonds (red), viewing down the two-fold symmetry (**d**), three-fold symmetry (**e**) and four-fold symmetry axes (**f**).

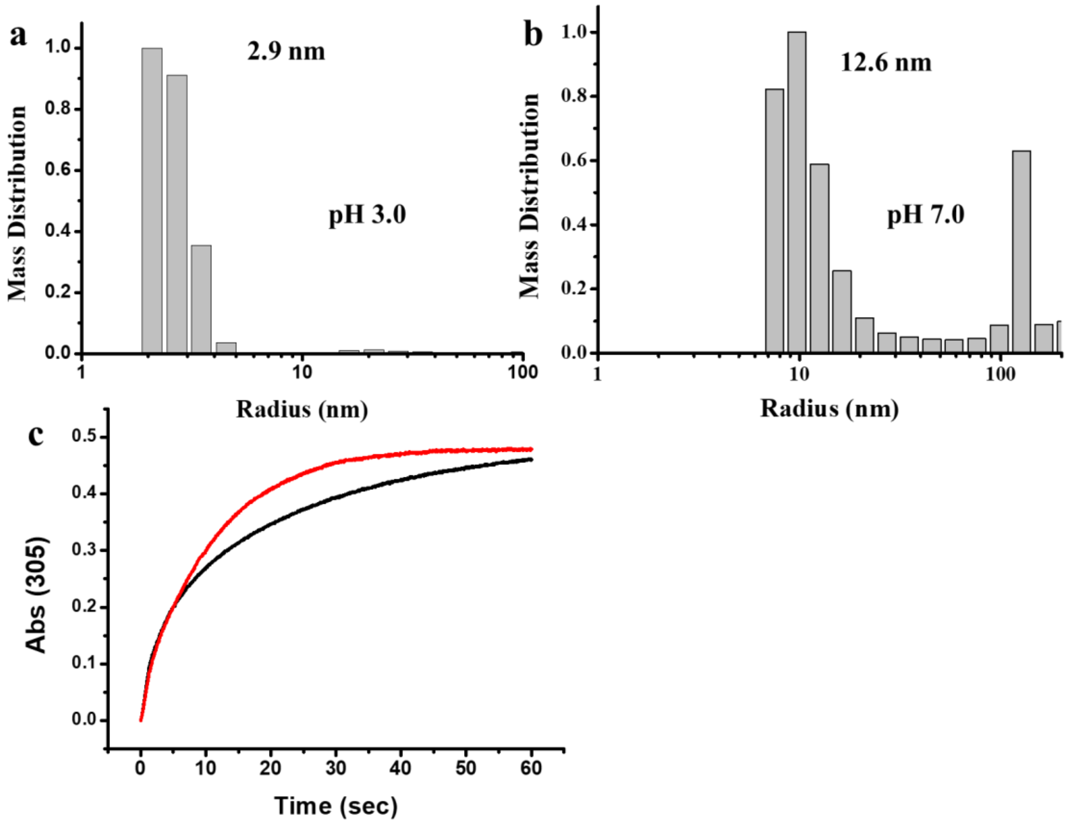

Supplementary Figure 19. Characterization of 48-mer. DLS analyses of the 48-mer protein nanocage in the presence of Mg^{2+} at pH 3.0 (**a**) and 7.0 (**b**). Protein association takes place with the 48-mer protein nanocage at 7.0, which is derived from its larger protein surface as compared to its analogues such as the 24-mer and 16-mer protein nanocages. When pH decreases to 3.0, protein nanocages disassemble into subunits (**a**). (**c**) Kinetic curves of Fe^{2+} oxidation by O_2 in the presence of native HuHF (red line) and 48-mer (black line), respectively.

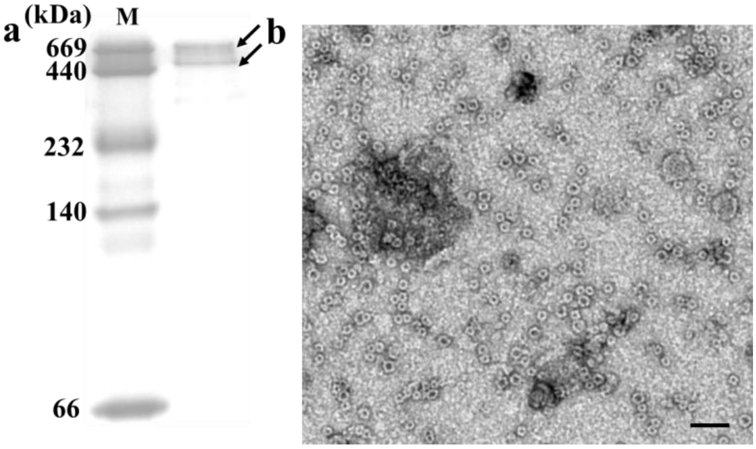

Supplementary Figure 20. Mg^{2+} facilitates the formation of 48-mer$_{\Delta 3C\text{-}\nabla C}$ during cell culture. (**a**) Native-PAGE analyses of overexpressed protein products upon *E. coli* expressing mutant Δ3C-∇C treated with extra magnesium salts. Source data are provided as a Source Data file. (**b**) TEM image of overexpressed protein products upon *E. coli* expressing mutant Δ3C-∇C upon treated with extra magnesium salts. Scale bars represent 50 nm.

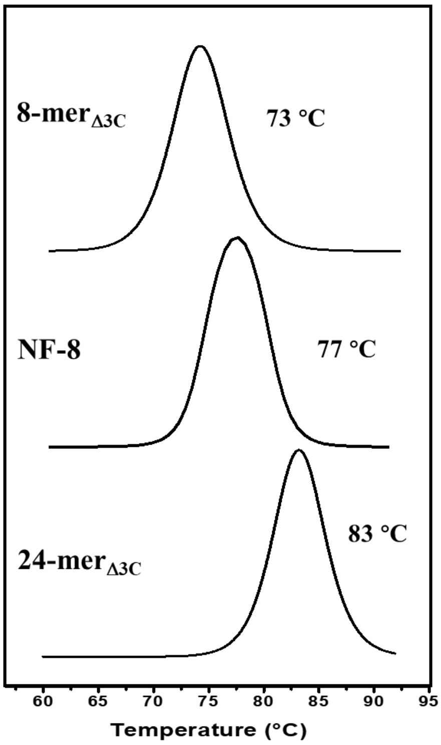

Supplementary Figure 21. DSC analyses of 8-mer$_{\Delta 3C}$, NF-8 and 24-mer$_{\Delta 3C}$. Disulfide bond and different assembly leads to the difference of melting points.

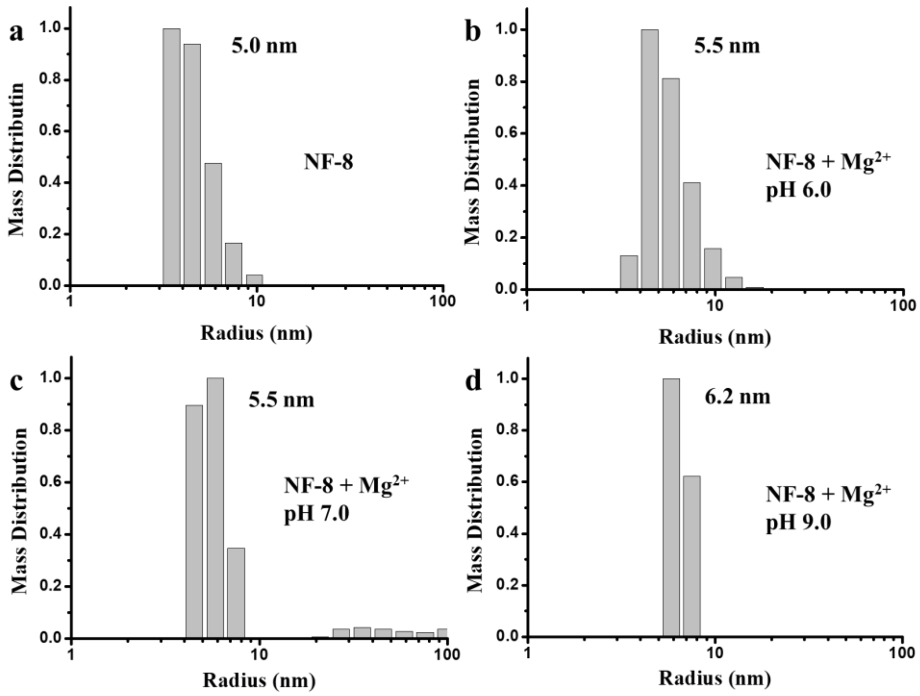

Supplementary Figure 22. Dynamic light scattering analyses of the 8-mer protein. One population with a R_H value of 5.0 nm is evident in the mass distribution curve of NF-8 alone (**a**), which corresponds to NF-8 protein monomer. After NF-8 was treated with Mg^{2+} at pH 6.0 (**b**), 7.0 (**c**), and 9.0 (**d**), there is also one population occurring in solution, the R_H of which is 5.5 nm, 5.5 nm, and 6.2 nm, respectively, these values being similar to that of NF-8 alone. These results demonstrated no protein assembly for NF-8 in the presence of Mg^{2+} *in vitro*.

Supplementary Tables

Supplementary Table 1 Crystallization conditions for each crystal.

	Crystallization conditions	pH
**24-mer$_{Δ3C}$**	1260 mM $(NH_4)_2SO_4$ and 100 mM HEPES/NaOH	7.5
**8-mer$_{Δ3C}$**	10% PEG 6000, 5% MPD and 100 mM HEPES/NaOH	7.5
**24-mer$_{Δ3C\text{-}∇C}$**	200 mM $MgCl_2$, 3.4 M 1,6-Hexanediol and 100 mM TRIS/HCl	8.5
**48-mer$_{Δ3C\text{-}∇C①}$**	200 mM NaCl, 35% MPD and 100 mM TRIS/HCl	8.5
**48-mer$_{Δ3C\text{-}∇C②}$**	200 mM $MgCl_2$, 30% PEG 400 and 100 mM TRIS/HCl	7.0

Supplementary Table 2 Crystallographic properties and data collection and model refinement statistics.

**Parameters**	**24-mer$_{\Delta 3C}$**	**8-mer$_{\Delta 3C}$**	**24-mer$_{\Delta 3C-\nabla C}$**	**48-mer$_{\Delta 3C-\nabla C①}$**	**48-mer$_{\Delta 3C-\nabla C②}$**
Wavelength (Å)	0.9779	0.9779	0.9779	0.9779	0.9779
Space group	*F432*	*P1*	*P12₁1*	*F432*	*F432*
Resolution range (Å)	40.66-3.104	49.16-4.443	40.87-3.868	48.05-2.699	45.46-2.998
Unit cell	181.844,181.844, 181.844, 90,90,90	52.304,139.223,139.114, 90.102,90.278,90.181	117.38,165.364,164.865, 90,94.002,90	235.374, 235.374, 235.374,90,90,90	236.229,236.229, 236.229,90,90,90
Total reflections	2056602	732485	3809615	5721563	5690958
Unique reflections	5057 (495)	21483 (1562)	54320 (4634)	15937 (1549)	11867 (1144)
Completeness (%)	99	87	92	100	100
Mean I/sigma (I)	4.7	2.3	2.7	8.1	6.5
Wilson B-factor	58.02	127.47	66.40	38.88	58.73
CC1/2	0.996	1.001	0.921	1.004	0.999
Reflections used in	5037 (495)	21166 (1538)	54283 (4630)	15934 (1549)	11865 (1144)
Reflections used for	263 (24)	882 (58)	2730 (243)	792 (83)	565 (65)
R-work	0.1971 (0.2199)	0.2066 (0.2742)	0.2432 (0.2497)	0.1998 (0.2654)	0.1914 (0.2423)
R-free	0.2750 (0.3190)	0.2761 (0.4367)	0.3279 (0.3671)	0.2435 (0.3681)	0.2456 (0.3189)
non-hydrogen atoms	1367	20155	32527	2550	2515
macromolecules	1363	20122	32516	2496	2494
Ligands	1	16	0	1	3
Protein residues	166	2440	3984	303	303
RMS (bonds)	0.011	0.005	0.014	0.009	0.009
RMS (angles)	1.24	0.81	1.54	1.00	0.96
Ramachandran	95	87	81	94	94
Ramachandran	2.4	9.5	14	5.4	4.7
Ramachandran	3	3.2	5.3	1	1
Rotamer outliers (%)	1.4	6.5	3.7	5.6	4.5

Supplementary Table 3 Primer sequences of NF-8 mutants.

Site	Primer sequence
**C90A**	5′GACATCAAAAAACCGGACGCGGATGATTGGGAATCTGGT
	3′ACCAGATTCCCAATCATCCGCGTCCGGTTTTTTGATGTC
**C102A**	5′GGTCTGAACGCAATGGAAGCAGCGCTGCATCTGGAGAAA
	3′TTTCTCCAGATGCAGCGCTGCTTCCATTGCGTTCAGACC
**C130A**	5′AAAAACGATCCGCATCTGGCCGATTTCATCGAAACCCAC
	3′GTGGGTTTCGATGAAATCGGCCAGATGCGGATCGTTTTT
**D144C**	5′CTGATCAAAGAACTGGGCTGCCACGTTACCAATCTGCGT
	3′ACGCAGATTGGTAACGTGGCAGCCCAGTTCTTTGATCAG
